# A Possible Case of Supraventricular Tachycardia Associated With a Ketone-Based Weight-Loss Supplement

**DOI:** 10.7759/cureus.71577

**Published:** 2024-10-15

**Authors:** Kerron Glean, Mehul Chawla, Ria Mahabir-Glean, Stefan Ganga, Kishan Ramsaroop

**Affiliations:** 1 General Internal Medicine, University Hospitals Plymouth NHS Trust, Plymouth, GBR; 2 Cardiology, South West Regional Health Authority, San Fernando, TTO

**Keywords:** arrhythmia, diet, ketones, supraventricular tachycardia, tachyarrhythmia, vigorous exercise, weight loss

## Abstract

Weight loss is well known to improve cardiovascular health. Its benefits are widespread including reducing cardiovascular morbidity and mortality, improving clinical outcomes in diabetic and hypertensive patients and mobility in osteoarthritis patients. A well-planned weight-loss program has many benefits; however, one should be careful when trying to lose weight too rapidly, especially when that involves radical weight-loss products or regimes. We present a possible case of supraventricular tachycardia (SVT) associated with a ketone-based weight-loss product in an overweight but otherwise healthy 44-year-old Indo-Caribbean male. The patient presented to the Emergency Department with palpitations and an ECG demonstrating SVT at 221 beats per minute (BPM). He was converted to sinus rhythm with adenosine, started on beta-blockers, and treated with intravenous fluids, which led to a positive outcome. This patient had no major risk factors for SVT development other than starting an intense weight-loss regime with a ketone-based weight-loss product a few days before the development of the tachyarrhythmia. Our aim is to explore the possibility of this ketone-based weight product contributing to the development of SVT in our patient and to discuss the implications of these products on cardiac health.

## Introduction

Supraventricular tachycardia (SVT) is a type of narrow complex tachycardia [1]. There is usually an abrupt onset and offset with a regular ventricular response [1]. There is a prevalence of 2.25/1000 persons and a female predominance (2:1) [2]. Although stimulants, thyroid, and cardiac disease are common causes of SVT, in many cases no identifiable cause is found. Obesity is a risk factor that can increase a patient's risk of arrhythmia development; however, in a study by Rina, et al., this risk was associated mainly with atrial fibrillation and not SVT in general [3]. Patients who present with SVT should be thoroughly investigated for a possible underlying cause in all cases. We present a novel case of the development of SVT, which may have been associated with a ketone-based weight-loss supplement. While there is literature that links ketones and the development of arrhythmias, there are no current case reports of this in vivo. Our patient was a previously healthy Indo-Caribbean male with a BMI of 29.5 who developed SVT a few days after beginning a ketone-based weight-loss supplement. He was cardioverted with adenosine in the emergency department and a single identifiable cause was not found. Our aim is to explore the possibility of this ketone-based weight product contributing to the development of SVT in our patient and to discuss its implications on cardiac health. Although weight loss has many benefits, we believe that patients who intend to do so rapidly or use weight-losing supplements should seek medical advice before proceeding as it can have a major effect on cardiovascular health. Ideally, weight loss should be done progressively over time with the advice of an expert such as a dietician, fitness trainer, or medical practitioner. 

## Case presentation

**Figure 1:**
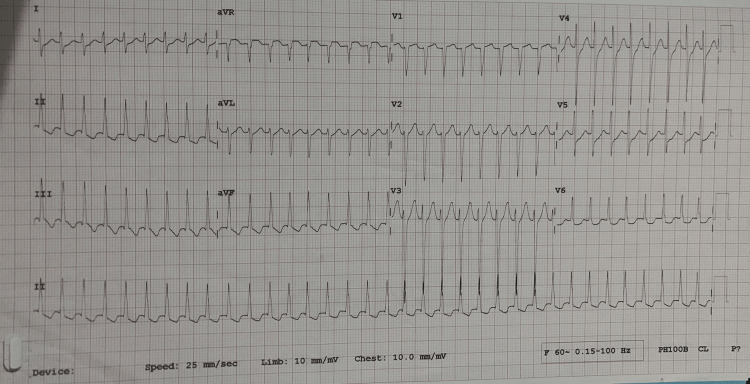
ECG showing initial tachyarrhythmia seen on admission to the hospital

An overweight 44-year-old Indo-Caribbean male with no other medical or surgical history presented to the emergency department at 2:15 am with palpitations, chest pain, dyspnea, and dizziness for approximately two hours. Cardiac monitoring revealed a narrow-complex tachycardia with a rate of 221 BPM. An ECG confirmed the patient had SVT, specifically atrioventricular nodal re-entry tachycardia (AVNRT) (Figure 1). 24 mg of adenosine was required to cardiovert the patient back to sinus rhythm with a rate of 92 beats per minute (BPM). Of note the patient had no delta waves present on his ECG. He was then referred to Cardiology and admitted to the ward. 

The patient had no prior chronic medical disease apart from being overweight with a BMI of 29.5. The patient informed the medical staff that he had recently begun an intense weight-loss program in the previous five days. This included going to the gym to do numerous cardiovascular and weightlifting exercises for three out of the prior five days, starting a whey isolate protein shake and a ketogenic weight-loss supplement of which he had taken three doses each. Of note, he had not exercised within 24 hours of admission to the hospital.

Otherwise, his drug history was negative and he denied illicit substance use. Family history was significant for a father who had a myocardial infarction at age 60. The patient had a 20-pack-year smoking history and would drink alcohol rarely. He denied the use of energy drinks, but he regularly consumed one cup of coffee daily and did not exceed that amount more than 24 hours before the presentation. 

Routine bloods including complete blood count (CBC), urea, electrolytes, and thyroid function tests (TFTs) were mostly unremarkable (Table 1). His troponin was mildly elevated at 120 ng/L, which was likely due to the tachycardia as a subsequent echocardiogram demonstrated an ejection fraction greater than 70% without any structural abnormalities or valvular disease. He was incidentally found to have rhabdomyolysis as well, which was a likely consequence of recent strenuous physical activity. He had a creatine kinase of 10,000 U/L and his urinalysis showed microscopic hematuria; however, urine microscopy did not reveal red blood cells and was otherwise unremarkable. The patient also had deranged liver function tests with an AST of 386 IU/L and an ALT of 171 IU/L. A triglyceride level of 685.9 mg/dL and cholesterol level of 220 mg/dL were also found. HIV and Hepatitis B and C titers were negative, but an abdominal ultrasound subsequently revealed undiagnosed metabolic dysfunction-associated steatotic liver disease. 

**Table 1:** Patient's initial blood test results on admission to hospital

TEST	RESULT	UNITS	REFERENCE RANGES
SODIUM	140	mmol/L	135 - 145
POTASSIUM	4.9	mmol/L	3.5 - 5.1
CHLORIDE	102.8	mmol/L	97 - 110
UREA	14	mg/dL	6 - 23
CREATININE	0.8	mg/dL	0.5-1.0
C-REACTIVE PROTEIN	0.200	mg/dL	0.1 - 0.5
CHOLESTEROL	220	mg/dL	170 - 200
TRIGLYCERIDES	685.90	mg/dL	40 - 160
CALCULATED LOW-DENSITY LIPOPROTEIN	49.82	mg/dL	40 - 130
ASPARTATE AMINOTRANSFERASE	386	IU/L	5.0 - 40
ALANINE AMINOTRANSFERASE	170.9	IU/L	5 - 41
TOTAL BILIRUBIN	0.24	mg/dL	0 - 1.20
DIRECT BILIRUBIN	0.11	mg/dL	0 - 0.40
INDIRECT BILIRUBIN	0.13	mg/dL	0 - 1
CREATINE KINASE	10,000	U/L	39 - 308
LACTATE DEHYDROGENASE	769	U//L	135 - 214
HIGH SENSITIVITY TROPONIN	0.12	ng/mL	0 - 0.1

He was treated with beta-blockers, rigorous intravenous fluids due to the elevated creatine kinase, prophylactic low molecular weight heparin, and low dose aspirin in the first instance while we excluded an acute coronary syndrome. He was subsequently discharged on a beta-blocker alone, with follow-up appointments scheduled with an electrophysiologist to further investigate his tachyarrhythmia and with gastroenterology for the follow-up of his fatty liver disease.

He was initially switched to flecainide by the electrophysiologist, and ablation therapy was planned; however, he declined and has not had a recurrence of SVT since stopping the ketone-based weight-loss supplement and the whey protein isolate shake. Interestingly, he informed the medical team subsequently that his cousin also developed SVT shortly after starting the same ketone-based weight-loss supplement. 

## Discussion

SVTs are characterized based on their site of reentry [1]. Atrioventricular nodal reentrant tachycardia (AVNRT) accounts for 60% of cases and is due to the formation of a reentry circuit confined to the AV node and perinodal atrial tissue. Our patient's ECG showed an AVNRT. Atrioventricular reentrant (or reciprocating) tachycardia (AVRT) accounts for 30% of cases and is due to an AV accessory pathway, linked by common proximal (the atria) and distal (the ventricles) tissues. Sinoatrial nodal reentrant tachycardia (SANRT) and AT can account for approximately the remaining 10% [1]. The diagnosis is made via ECG. The hemodynamic status of any patient with an SVT must first be established. A hemodynamically unstable patient with a narrow complex tachycardia may require DC cardioversion; if an SVT is diagnosed in a hemodynamically stable patient, the Valsalva maneuver and carotid massage may be attempted first. If the tachycardia does not resolve, adenosine may then be administered. Other drugs such as calcium channel blockers or beta-blockers may be used if there is no resolution with adenosine [2]. Although stimulants, thyroid, and cardiac disease are common causes of SVT, in many cases no identifiable cause is found. The patient should be thoroughly investigated for a possible underlying cause. These investigations include a CBC, basic metabolic profile (BMP), TFTs, echocardiogram, Holter monitor, and cardiac enzymes. If an underlying cause is found, then it should be treated; otherwise, the patient should be managed expectantly and counseled to avoid possible triggers [2]. 

The patient, like many other people, embarked upon a weight-loss regime that he did not adequately research. He gave his body no time to prepare or adjust before making drastic changes to his diet and activity without appropriate monitoring. The patient was overweight with a BMI of 29.5. While he does not officially fall into the category of obesity, a study by Rina, et al. shows that obesity is an independent risk factor for the development of arrhythmia [3]. While this study demonstrates an increased risk of developing atrial fibrillation by 50%, it does not specifically state that this risk is translated to all tachyarrhythmias.

He began taking ketone-based weight-loss supplements, which contained magnesium beta-hydroxybutyrate, sodium beta-hydroxybutyrate, calcium beta-hydroxybutyrate, and caffeine anhydrous. Although the increased caffeine intake may have increased the risk of SVT development, there have been studies that also show an association between ketones and arrhythmogenesis [4]. 

A study by Best, et al. involving children on ketogenic diets showed prolonged QT interval (QTc) and dilated ventricles in some cases, which normalized when the diet was discontinued [5]. Another study by Kalra, et al., however, showed modern ketogenic diets followed with attention to electrolyte and mineral balance have been found to be safe and were not associated with arrhythmias. This shows that these diets can be safely undertaken with appropriate monitoring. It also showed that electro-cardio vigilance is required while initiating and continuing a ketogenic diet [6]. Both studies showed that a noteworthy association between prolonged QTc and both low-serum bicarbonate and high-beta-hydroxybutyrate had been observed [5,6]. This association can be substantiated by studies that have shown increased membrane excitability in association with ketone bodies [4]. There have also been experimental studies that postulate a possible correlation between cardiomyopathy and decreased ketone body oxidation in the myocardium [3]. The data in these cases is limited, however, and requires further study.

## Conclusions

While our patient is overweight, which can increase his risk of arrhythmogenesis, the studies that link increases in ketones in the body with cardiac instability provide a different perspective on the possible cause of the development of his SVT. It is important to reiterate that this patient has not had a recurrence of any tachycardia while being off anti-arrhythmic treatment since his initial presentation. His BMI and lifestyle remain the same; the only change he has made is that he has stopped taking protein whey isolate and ketone-based supplements. While it is difficult to definitively attribute causality, the studies showing an association between ketones and arrhythmogenesis raise the question of whether the use of the ketone-based supplement played an integral role in the development of SVT in the patient. As such, we believe that further study into the effects of ketone-based products on the heart should be considered.

While exercise and weight loss provide well-documented physical and mental health benefits, they should be done safely with expert advice to prevent the possible deleterious outcomes that the patient suffered, especially if ketogenic weight-loss products are used. This is why we recommend that patients with risk factors for cardiac disease should consult their physician before the initiation of such weight-loss products.
